# Open Tibial Fracture in a Non-Compliant Patient: A Case Report

**DOI:** 10.3390/jfmk3030044

**Published:** 2018-08-11

**Authors:** Samuele Pizzolo, Gianluca Testa, Giacomo Papotto, Giuseppe Mobilia, Giovanni Di Stefano, Giuseppe Sessa, Vito Pavone

**Affiliations:** Department of General Surgery and Medical Surgical Specialties, Section of Orthopaedics and Traumatology, AOU Policlinico-Vittorio Emanuele, University of Catania, Via Plebiscito 628, 95100 Catania, Italy

**Keywords:** open tibial fracture, Gustilo–Anderson classification, compliance, intramedullary nailing, external fixation

## Abstract

Open tibial fractures represent the most frequent fractures of long bones, comprising approximately 1.9% of all fractures. Although locked intramedullary nailing is the gold standard for treating closed and unstable tibia diaphyseal fractures, for most exposed fractures, an external fixator can first be used, followed by conversion through an intramedullary nail. The present report describes the case of a 17-year-old male who presented with a complex multi-segmented displaced tibia fracture, type 42-C3, with exposure of IIIB type according to the Gustilo–Anderson classification, and with an attached disrupted fracture of peroneal malleolus, type 44-B2. External fixation was the preferred treatment method. Before the definitive surgical treatment, the patient had a second accident that caused refracture and damage to the soft tissues and external fixation system. This prolonged the time estimated for the conversion from the external fixator to the intramedullary nail. The reported case shows the use of various treatment steps with different timelines and an intervention with vacuum-assisted closure therapy for soft tissue healing as well as subsequent intramedullary nailing in order to reach the definitive healing of a non-compliant patient. These combined methods achieved an acceptable reduction and good stability of such a complex fracture.

## 1. Introduction

Shaft tibial fractures are relatively common, yet they are complex to manage, due to poor soft tissue coverage on the antero-medial surface of the tibia and possible alterations of alignment and stability of the lower limb. This may result in bone and soft tissue injuries, leading to exposed fractures and to non-negligible complications, such as infections and delayed healing or non-unions [1].

These fractures often result from direct, high-energy traumas, such as road, work, or sports injuries. They frequently affect young adults with an average age of 37 years. The frequency of tibial fracture is approximately 11–26 fractures per 100,000 per year, with open fracture being 1.9% of all tibial fractures [2,3]. Cases of indirect trauma, such as those due to falling long distances, are less common.

The most complete classification of diaphyseal tibial fractures is the AO/OTA (Orthopedic Trauma Association) classification, which is based on standard radiographic projections and includes the fracture site as well as the degree and type of comminution [4]. It distinguishes between simple fractures (A), compression fractures (B), and complex fractures (C). Each group is then further divided into three subgroups (Table 1). Type A fractures are mono-focal, and their subdivision into the various subgroups is based on the orientation of the fracture and the presence or absence of a fibular fracture, resulting in the following categories: spiroid fractures [A1], oblique fractures [A2], and transverse fractures [A3]. The suffix (1) is used if there is no associated fracture of the fibula, (2) is used if the peroneal fracture is distant from the tibial fracture, and (3) is used if it is at the same level. In type B fractures, the subdivision is very similar, including spiroid wedge fractures [B1], flexed wedge fractures [B2], and fragmented wedge fractures [B3]. Type C fractures are not divided according to the position of the fibula fracture relative to the tibial fracture but are based on the severity of the tibial fracture and classified as complex spiroid fractures [C1], bifocal fractures [C2], and comminuted fractures [C3].

Open fractures are subdivided according to the Gustilo–Anderson classification [5]. This classification is based on soft tissue damage, and grade III considers a variety of different fractures on the basis of their morphology, extent of contamination, damage to soft tissue, or delayed treatment. Grade III sub-categories are identified on the basis of periosteal damage and the need for vascular surgery (Table 2).

Surgical intervention aims to obtain good correction and reduction of the fracture, an adequate axial alignment on the sagittal and coronal planes, a suitable range of motion, the restoration of the correct length of the limb, as well as good and early knee and ankle motility.

To evaluate the clinical and radiographic outcomes as well as the complex management of these fractures, we report a clinical case of an open-shaft fracture of the tibia with an associated fracture of the peroneal malleolus in a 17-year-old uncooperative patient.

## 2. Clinical Case

**Figure 1:**
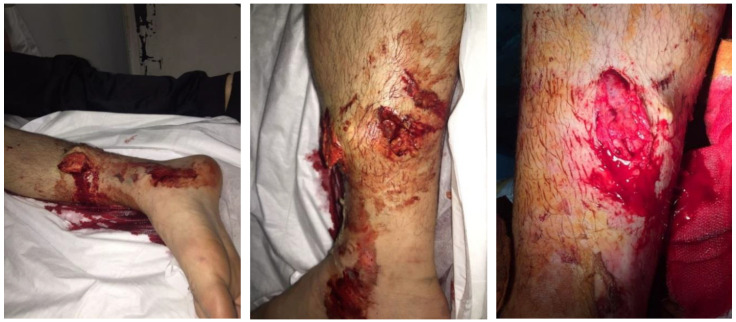
Degree of exposure of the fracture and soft tissue at the level of the right leg.

In June 2016, a 17-year-old boy came to our attention after being referred to us following a motorcycle accident. Informed consent from the patients was obtained. He immediately appeared non-compliant with healthcare workers. The first clinical evaluation revealed important and generalised pain of the right leg as well as functional impotence, tibial fracture with medial exposure of degree IIIB of about 10 cm of extension at the level of the distal middle third of the right tibia, contamination of the same outbreak with material that probably originated from the road surface, and various lacerated contusion wounds spread around the exposure (Figure 1).

**Figure 2:**
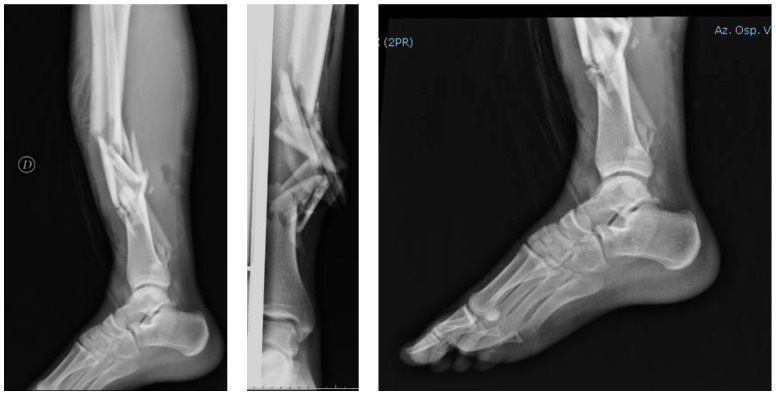
Pre-surgical emergency radiographs; anteroposterior (AP) and left lateral (LL) projections of the right leg and ankle.

The patient showed no apparent deep vascular-nervous deficits and reported being a habitual smoker. After appropriate evaluation by the general emergency room surgeon in order to rule out abdominal and/or cranial lesions, the patient was sent to radiology to perform anteroposterior and lateral radiographs, which showed a complex multi-segmented displaced tibia fracture (type 42-C3) with an attached disrupted fracture of peroneal malleolus, type 44-B2 (Figure 2).

**Figure 3:**
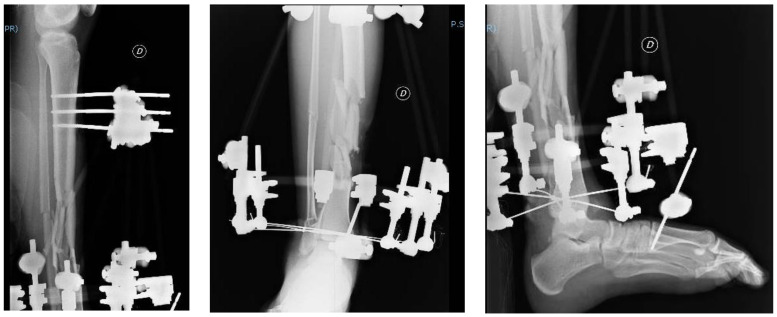
Post-operative radiographs; AP and LL projections of the right leg and ankle.

The patient was surgically treated with a reduction and stabilization with external fixation. The patient was placed in the supine position, and the exposure was debrided to remove the contaminating residues. Various bruised wounds and bruises were thoroughly cleaned. The fracture was reduced by mounting an external fixation implant in a hybrid configuration with a tibial socket of three proximal Schanz screws and three distal 2 mm transverse wires anchored to a semicircular frame (Figure 3).

**Figure 4:**
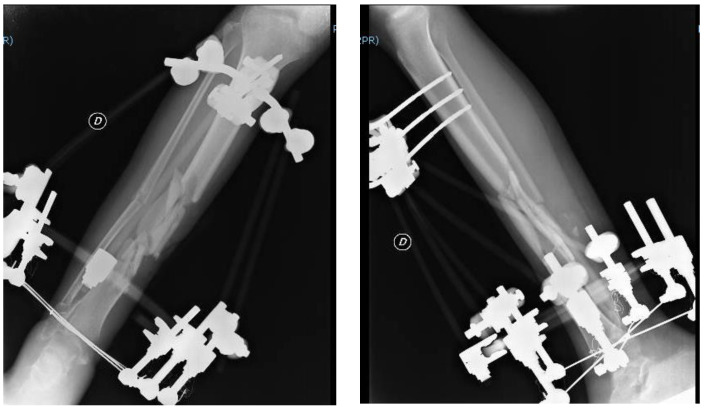
Radiograph at the three-month follow-up evaluation; AP and LL projections of the right leg and ankle.

One month later, the patient underwent a clinical and radiographic follow-up evaluation, which showed an implant with good grip and a reduction of the fracture abutments. The patient underwent new clinical and radiographic checks two months and three months after his surgery (Figure 4). The multi-fragmentation of the fracture still showed no stable consolidation during these controls, with poor presence of bone callus; conversion to intra-medullary nailing had been planned at soft tissue healing.

**Figure 5:**
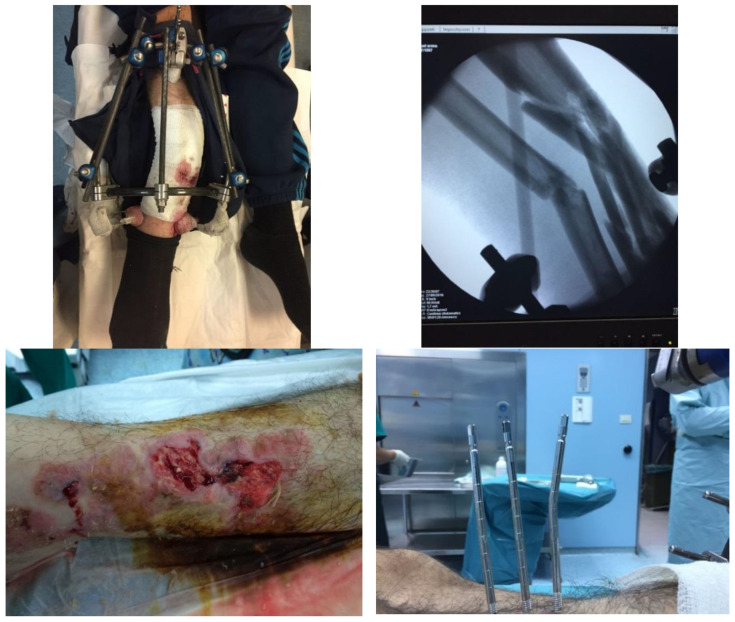
Images of the fracture, soft tissue, and external fixation implant following the new motorcycle accident three months after the first one.

Three months after surgical treatment, the patient suffered a new motorcycle accident in which he reported the refracture and decomposition of the anatomical axis and the abutments, deformity of the anatomical profile, and damage to the soft tissues and external fixation system (Figure 5).

**Figure 6:**
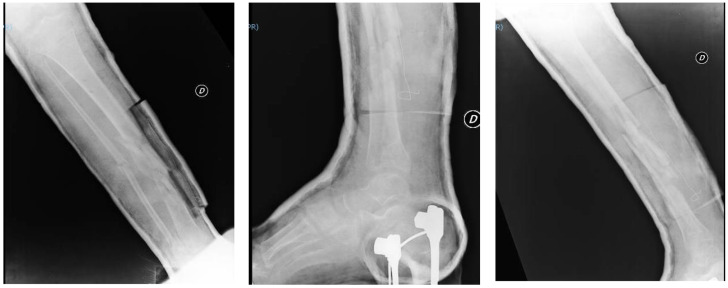
Radiograph of the right leg and ankle after the new treatment performed in October 2016, including the removal of the external fixator and packaging of the pin-femoral-podalic appliance incorporating a trans-calcaneal traction bracket.

A new surgical treatment was performed. The external fixator was removed, the fracture abutments were reduced, and a long cast with a trans-calcaneal traction was performed. The cast was anteriorly open because the tibial fracture focus was prominent at the level of the soft tissue, encompassing the trans-calcaneal traction bracket (Figure 6).

**Figure 7:**
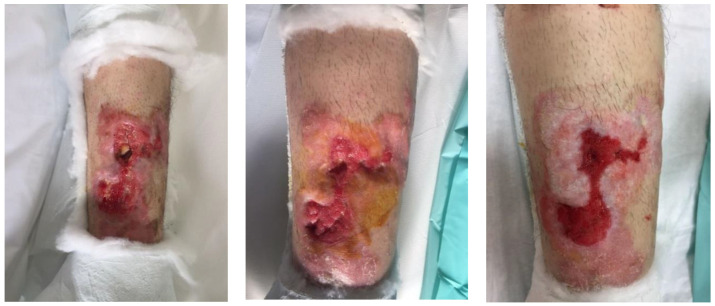
Condition of progressive improvement of soft tissues following vacuum-assisted closure (VAC) therapy.

In the following days, vacuum-assisted closure (VAC) therapy was started to allow the progressive healing of the soft tissues overlying the tibial fracture focus (Figure 7).

**Figure 8:**
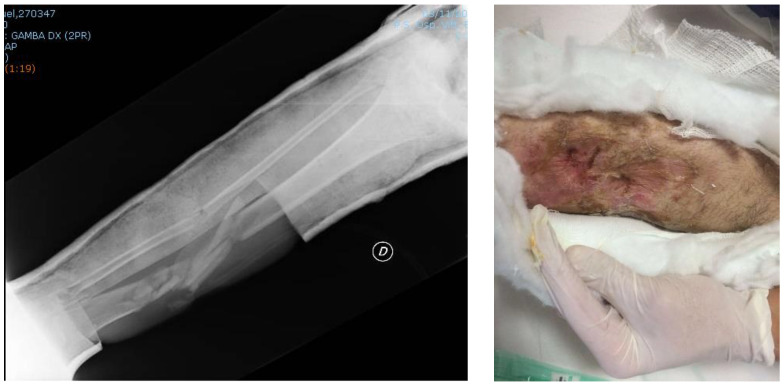
Radiograph of the right leg and image of the soft tissue in November 2016.

At the visit carried out in November 2016, the patient’s soft tissues had significantly improved, while the fracture was basically unchanged and not fully healed (Figure 8). 

**Figure 9:**
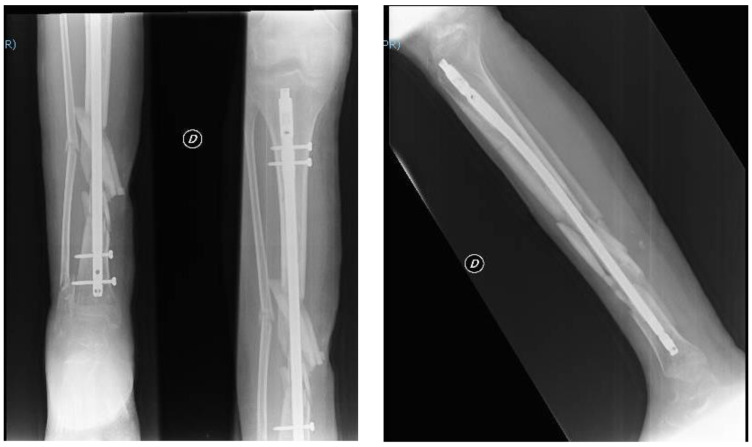
Postoperative radiograph of the right leg performed in December 2016.

After 45 days with a plaster cast, a decision was made to perform a definitive surgical treatment. This involved intramedullary nailing blocked with two proximal screws and two distal screws to realign and reduce the tibial and peroneal fractures (Figure 9).

**Figure 10:**
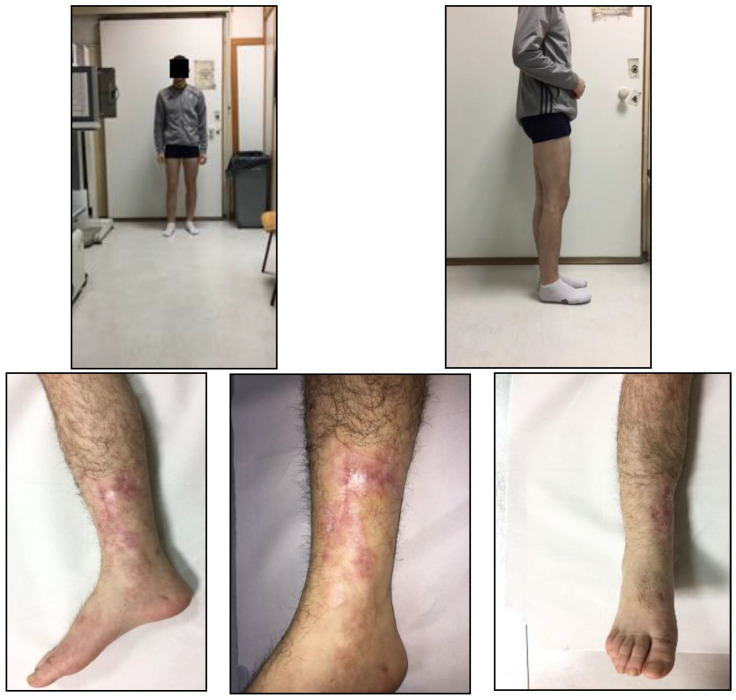

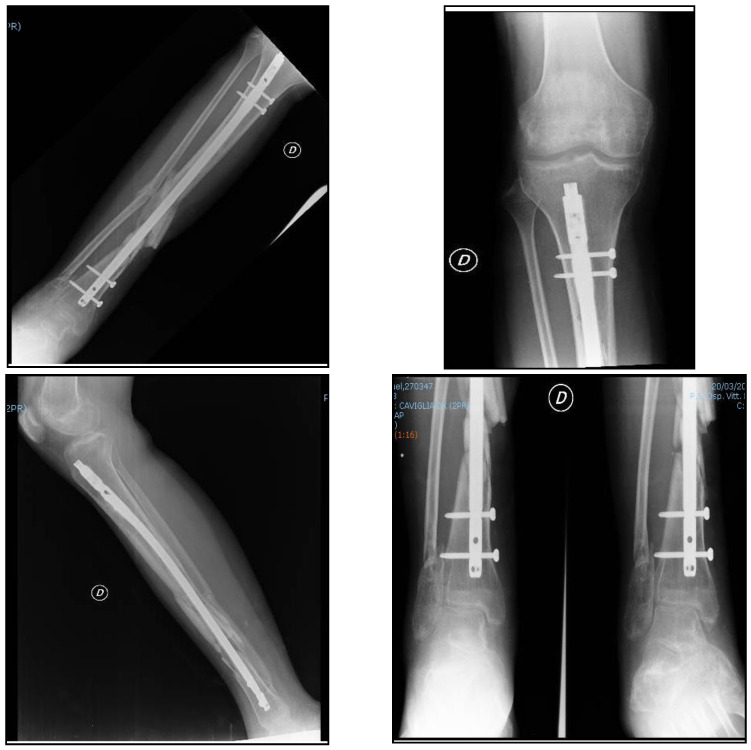
Clinical-radiographic follow up in February 2017.

Subsequent monthly and two-monthly clinical-radiographic follow-up evaluations showed progressive healing of the tibia and fibular fractures and improvement of the functional clinical prognosis (Figure 10).

### Post-Operative Care

Such fractures can lead to a significant loss of functionality and may limit the ability to walk, run, and participate in sports and work. Progressive loading on the lower right limb was possible after about 15 days after the surgical nailing operation. This was performed to stimulate bone healing, while remaining compatible with the expressed tolerance of the patient.

To evaluate the results, we considered both clinical and radiological criteria. We performed the last and accurate objective examination and radiographic follow-up evaluation in February 2017, two months after the intramedullary nailing surgery, which showed sufficient knee and ankle joint motion with an initial consolidation of the fracture. Physiotherapy played an important role in recovering the normal joint function. The initiation of physiotherapy often varies, depending on the type of surgery performed and the severity of the fractures and damage to soft tissues.

A rehabilitation consisting of passive and active muscular mobilisation exercises was carried out to assist functional joint recovery as well as the maintenance of posture in orthostatism, balance, proprioception, adequate tension, and muscular strength. All of these were limited and did not allow for normal daily activities, following the long period of immobilization. The muscles were strengthened and affected the lower limb from the hip to the ankle. In the first period (after about 30 days), isometric toning exercises were performed by contracting the limb at one-minute intervals. Balance exercises were performed on the injured limb, toning exercises were performed using axial compression, and flexion exercises were performed by extending the foot and leg toward the thigh. At the beginning of rehabilitation, the use of crutches was necessary. In general, with progressive walking and balance improvement, patients may only require one crutch before they can move and walk freely, according to the patient’s tolerance. The walking style, amplitude of movement, strength, swelling, pain, and surgical scars were all taken into consideration. Ice-based therapies, electrical stimulation, and heat were applied to reduce pain and swelling and to allow for earlier bone healing. Swimming and cycling helped with muscle recovery. Also, lifting exercises and lowering of the affected leg were performed to assist the recovery of the quadriceps femoris and the gastrocnemius.

Some rehabilitation treatments were performed at home after being introduced by a physiotherapist. Massages and scar tissue mobilisation were performed. The recovery times vary for each patient and can only be approximately calculated. They mostly depend on the conditions at the start of rehabilitation after surgery. Physiotherapy for fracture cases similar to the present one usually lasts 4 to 10 weeks. In the presented case, physiotherapy lasted about seven weeks until the recovery of mobility and return to normal activities were safely achieved. Patient compliance is essential for good results, and, in this case, the patient was oftentimes non-compliant.

## 3. Discussion

External fixation is a widely used method to treat exposed tibia fractures, as it is considered to have a low incidence of infections. It does not require the placement of devices on the fracture site and is characterised by a lower risk of vascular damage. It can also be used as a definitive or temporary fixation. The external fixations allow for the use of multi-planar mounts—with medial and lateral bars fixed by screws or by piercing pins—in quadrilateral, triangular, or other particular configurations, depending on which method provides the best possible stability [6].

Circular external fixators [7] and hybrid fixators exhibit the highest configuration complexity. Circular fixators are comprised of complete rings or semi-rings that are fixed to the tibia by tensioned piercing threads. Hybrid fixators are comprised of a system of both rings with tensioned piercing wires and bars with screws.

Locked intramedullary nailing is the gold standard for treating closed and unstable tibial shaft fractures and most exposed fractures (types I, II, and IIIA Gustilo–Anderson) [8]. Numerous studies have reported a high percentage of consolidation and a low incidence of incorrect consolidation and infections, even in cases of exposed fractures. If there is any doubt about the possibility of a shaft tibial fracture treatment with an intramedullary nail, it is possible to begin the initial treatment with an external fixation and then perform the definitive fixation with an intramedullary nail [9]. Contraindications to the initial nailing of a tibial shaft fracture include pre-existing deformities, open conjugation cartilage, a bone marrow channel less than 8 mm wide, wounds near surgical access, severe contamination, exposed fractures occurred more than 24 h before treatment, and type-IIIC Gustilo open fractures.

The reported case demonstrates the management of a patient with poor compliance that required the use of various treatment steps and adjusted timelines. A second motorcycle accident occurred and destroyed the external fixation implant, creating a refracture. Therefore, intervention with VAC therapy for soft tissue healing and subsequent intramedullary nailing was necessary. A cast combined with VAC therapy enabled soft tissue healing. Immediate nailing would not have been a prudent choice because of the patient’s highly critical conditions [10]. This decision allowed the functional recovery of the patient through early mobilisation and an appropriate rehabilitation process. This consisted of passive and active mobilisation exercises and muscle strengthening for the purpose of functional joint recovery. It also included orthostatism posture maintenance and improvements of balance, proprioception, tension, and muscle strength, which, following the long period of immobilisation, were limited and hindered normal daily activities.

## 4. Conclusions

The treatment of shaft tibial fractures is generally surgical, and there are typically multiple options. The gold standard is intramedullary nailing, which can be performed through various surgical techniques and is able to guarantee excellent results with relatively low complication rates.

External fixation is a widely used method in exposed tibia fractures, which has been shown to lead to a lower incidence of infections than intramedullary nailing and can be used as a temporary treatment method.

External fixation was the preferred option in this specific case. However, the patient’s poor compliance altered the timing originally estimated for the conversion from the external fixator to the intramedullary nail. This lengthened the treatment time and made soft tissue removal necessary using VAC Therapy, following the refracture and soft tissue injury that subsequently occurred.

Functional recovery through physiotherapy and progressive loading on the affected limb post-nailing had an essential role in this complex case. The physiokinetic therapy for similar fracture cases usually lasts 4 to 10 weeks; in this case, it took approximately seven weeks until mobility was recovered, and the patient was able to return to his normal activities. 

## Figures and Tables

**Table 1:** AO classification of tibial shaft fractures.

A1	A2	A3	B1	B2	B3	C1	C2	C3
Spiral	Oblique	Transverse	Spiral wedge	Oblique wedge	Transversal wedge	Comminuted	Segmental	Crush

**Table 2:** Gustilo–Anderson Classification of open fractures.

**I**	Open fracture, clean wound, wound <1 cm in length.
**II**	Open fracture, wound >1 cm but <10 cm in length without extensive soft tissue damage, flaps, avulsions.
**IIIA**	Open fracture with adequate soft tissue coverage of a fractured bone despite extensive soft tissue laceration or flaps, or high-energy trauma regardless of the size of the wound.
**IIIB**	Open fracture with extensive soft tissue loss and periosteal stripping and bone damage. Usually associated with massive contamination. Will often need a further soft tissue coverage procedure.
**IIIC**	Open fracture associated with an arterial injury requiring repair, irrespective of degree of soft tissue injury.
